# High overlap in patients diagnosed with hypermobile Ehlers-Danlos syndrome or hypermobile spectrum disorders with fibromyalgia and 40 self-reported symptoms and comorbidities

**DOI:** 10.3389/fmed.2023.1096180

**Published:** 2023-04-25

**Authors:** DeLisa Fairweather, Katelyn A. Bruno, Ashley A. Darakjian, Barbara K. Bruce, Jessica M. Gehin, Archana Kotha, Angita Jain, Zhongwei Peng, David O. Hodge, Todd D. Rozen, Bala Munipalli, Fernando A. Rivera, Pedro A. Malavet, Dacre R. T. Knight

**Affiliations:** ^1^Department of Cardiovascular Medicine, Mayo Clinic, Jacksonville, FL, United States; ^2^Division of Cardiovascular Medicine, University of Florida, Gainesville, FL, United States; ^3^Department of Psychiatry and Psychology, Mayo Clinic, Jacksonville, FL, United States; ^4^Department of General Internal Medicine, Mayo Clinic, Jacksonville, FL, United States; ^5^Department of Quantitative Health Sciences, Mayo Clinic, Jacksonville, FL, United States; ^6^Department of Neurology, Mayo Clinic, Jacksonville, FL, United States

**Keywords:** fibromyalgia, hypermobile Ehlers-Danlos syndrome (hEDS), hypermobility spectrum disorders, chronic disease, pain, abuse, allergy, women’s health

## Abstract

**Background:**

Joint pain is a common symptom in patients with hypermobile Ehlers-Danlos Syndrome (hEDS), hypermobility spectrum disorders (HSD) and fibromyalgia. The goal of this study was to determine whether symptoms and comorbidities overlap in patients diagnosed with hEDS/HSD and/or fibromyalgia.

**Methods:**

We retrospectively examined self-reported data from an EDS Clinic intake questionnaire in patients diagnosed with hEDS/HSD, fibromyalgia, or both vs. controls with an emphasis on joint issues.

**Results:**

From 733 patients seen at the EDS Clinic, 56.5% (*n* = 414) were diagnosed with hEDS/HSD and fibromyalgia (Fibro), 23.8% (*n* = 167) hEDS/HSD, 13.3% (*n* = 98) fibromyalgia, or 7.4% (*n* = 54) none of these diagnoses. More patients were diagnosed with HSD (76.6%) than hEDS (23.4%). Patients were primarily White (95%) and female (90%) with a median age in their 30s (controls 36.7 [18.0, 70.0], fibromyalgia 39.7 [18.0, 75.0], hEDS/HSD 35.0 [18.0, 71.0], hEDS/HSD&Fibro 31.0 [18.0, 63.0]). There was high overlap in all 40 symptoms/comorbidities that we examined in patients diagnosed with fibromyalgia only or hEDS/HSD&Fibro, regardless of whether they had hEDS or HSD. Patients that only had hEDS/HSD without fibromyalgia had far fewer symptoms/comorbidities than patients with hEDS/HSD&Fibro. The top self-reported issues in patients that only had fibromyalgia were joint pain, hand pain when writing or typing, brain fog, joint pain keeping from daily activities, allergy/atopy and headache. Five issues that significantly and uniquely characterized patients diagnosed with hEDS/HSD&Fibro were subluxations (dislocations in hEDS patients), joint issues like sprains, the need to stop sports due to injuries, poor wound healing, and migraine.

**Conclusion:**

The majority of patients seen at the EDS Clinic had a diagnosis of hEDS/HSD plus fibromyalgia that was associated with more severe disease. Our findings indicate that fibromyalgia should be routinely assessed in patients with hEDS/HSD and vis-a-versa to improve patient care.

## Introduction

Fibromyalgia is present in as many as 2–8% of the population worldwide depending on the diagnostic criteria (1, 2) and is the leading cause of chronic musculoskeletal pain accompanied by fatigue, sleep, and mood disturbances like anxiety and depression (2). The condition is multi-system and complex, leading to an average of 10 outpatient visits per year (3). Although the cause of fibromyalgia is not clear, it has a strong genetic basis revealed by family history and twin studies as well as environmental triggers (4, 5). Around 50% of patients report that their symptoms started after a particular event like an infection or trauma and it is hypothesized that fibromyalgia can be triggered by certain types of infections (e.g., Epstein-Barr virus, hepatitis C virus, HIV, Lyme disease) as well as physical and psychological trauma (e.g., motor vehicle collisions, post-traumatic stress disorder/PTSD after deployment to war) (2, 6–8). Central sensitization, which describes changes in the central and peripheral nervous system that increase sensitivity to painful and non-painful stimuli, is a key process in patients with fibromyalgia who often report widespread pain from childhood that gets worse with age (2, 9).

Hypermobile Ehlers-Danlos syndrome (hEDS) and hypermobility spectrum disorders (HSD) are connective tissue disorders that have a strong genetic basis from family history but as of yet no identified gene (10, 11). Hypermobile EDS/HDS is estimated to occur in 3% or 255 million people worldwide (12). Patients with hEDS/HSD have significantly greater joint and musculoskeletal pain, fatigue, anxiety, and depression than the general public (13, 14). Multiorgan comorbidities are common and can be debilitating (10, 12). It has been hypothesized that persistent nociceptive input due to damage to tissue from recurring joint instability in patients with hEDS/HSD may trigger central sensitization in dorsal horn neurons leading to widespread musculoskeletal pain (15–17).

Many symptoms and comorbidities are similar between hEDS/HSD and fibromyalgia, but only a few studies have examined whether the two conditions co-occur or whether symptoms and comorbidities overlap. Ofluoglu et al. compared 93 women diagnosed with fibromyalgia to 53 controls and found that hypermobility based on the Beighton score co-occurred in 64% of patients with fibromyalgia and 22% of controls (16). Similarly, Sendur et al. found that 46% of women diagnosed with fibromyalgia (*n* = 118) were hypermobile based on Beighton scores compared to 28% of controls (*n* = 118) (18). More recently Eccles et al. found that 81% of fibromyalgia patients (*n* = 64) were hypermobile based on Beighton scores but only 18% were diagnosed with hEDS based on the 2017 diagnostic criteria (17, 19). To our knowledge no studies have examined whether a large number of symptoms/comorbidities overlap in hEDS/HSD and fibromyalgia patients or defined the key characteristics that separate or connect patients with these conditions. For this reason, we embedded a diagnostic fibromyalgia questionnaire in our EDS Clinic intake questionnaire to determine the number of fibromyalgia patients being seen in the EDS Clinic that was then confirmed by a physician in-person (20). The goal of this study was to determine whether overlapping symptoms and comorbidities occurred in patients diagnosed with hEDS/HSD and/or fibromyalgia to determine whether the patients represent the same or distinct diagnostic groups to improve patient care.

## Materials and methods

### Ethics statement

Retrospective review of the demographic and clinical data from the medical records reported in this manuscript was approved by the Institutional Review Board (IRB# 19-011260) and informed consent was waived by the Institutional Review Board for all patients. The research conformed to the principles outlined in the Declaration of Helsinki.

### EDS Clinic data collection

Patient data were collected from December 2019 to October 2022 using a clinical 200-question REDCap intake questionnaire given to patients as standard of care prior to their first appointment at the EDS Clinic. Demographic and clinical data were extracted retrospectively from the intake questionnaire from patients that were 18 years or older at their first appointment. Patients seen at the EDS Clinic received a diagnosis of hEDS or HSD according to the 2017 diagnostic criteria (19). The Fibromyalgia Pain Assessment questionnaire with the diagnostic requirements from the 2016 Revisions of the 2010/2011 Fibromyalgia Diagnostic Criteria (21) was given to all patients as part of the intake questionnaire and was scored utilizing the calculations function in REDCap. The physician-based criteria from the 2016 Revisions are valid for individual patient diagnosis, whereas the self-report survey version of the criteria is not valid for clinical diagnosis in individual patients but is valid for research studies (21). In this study, patients identified as having fibromyalgia by the questionnaire were further assessed by a physician at the EDS Clinic for a formal in-person diagnosis of fibromyalgia. Patients with a hypermobility diagnosis of hEDS or HSD or a fibromyalgia diagnosis were extracted from the electronic medical record (EMR). In this manner we were able to distinguish patients with a hypermobile syndrome diagnosis (hEDS/HSD) and fibromyalgia (hEDS/HSD&Fibro), fibromyalgia only (Fibro), hEDS or HSD only (hEDS/HSD, hEDS or HSD), or patients without any of these diagnoses (control). Note that control patients came to the EDS Clinic because they had concern about having a connective tissue disorder, and so were not healthy controls but reported having chronic pain and many symptoms/comorbidities. We chose a sample of 40 self-reported symptoms/conditions from the 200 available in the EDS Clinic questionnaire focusing on joint issues, which are commonly reported in hEDS/HSD and fibromyalgia patients, as well as other symptoms/conditions that we often encounter in our EDS Clinic (directed by DRTK) and Fibromyalgia Clinic (directed by BKB) at Mayo Clinic Florida.

### Statistical analysis

Continuous variables were summarized with the sample median and range. Categorical variables were summarized with number and percentage of subjects. Kruskal–Wallis rank sum test was used to compare the difference of continuous variables among groups. Fisher’s exact test was used to examine the association between two categorical variables. *P*-values were adjusted for multiple testing using the method of Bonferroni. All the tests were two-sided and *p*-values < 0.05 were considered statistically significant. All statistical analyses were performed using R (version 4.1.2).

## Results

### Patient demographics

From 733 patients we identified that had been assessed for hEDS/HSD or fibromyalgia, we found that 56.5% (*n* = 414) were diagnosed with hEDS/HSD&Fibro, 23.8% (*n* = 167) hEDS/HSD without Fibro, 13.3% (*n* = 98) Fibro without hEDS or HSD, and 7.4% (*n* = 54) none of these diagnoses (controls). Demographic data were not significantly different between the groups for race (White *p* = 0.52, Black *p* = 0.059, Asian *p* = 0.20, American Indian/Alaskan native *p* = 0.72), ethnicity (*p* = 0.06), highest level of education (*p* = 0.14) or exposure to cigarette smoke (*p* = 0.59) (Table 1). Controls had fewer females (77%) (*p* = 0.002) and more males (22%) (*p* < 0.001), while all other groups were around 90% female and 95% White (Table 1). Although patients with hEDS/HSD&Fibro were almost a decade younger with a median age of 31.9 (18.0, 63.0) compared to Fibro patients who had a median age of 39.7 years (18.0, 75.0) (*p* < 0.001), all groups including controls had a median age in their 30s (Table 1).

**TABLE 1:** Demographics of female and male patients diagnosed at the EDS Clinic (*n* = 733).

Condition	Control (no fibro, hEDS or HSD) (*n* = 54)	Fibro (*n* = 98)	hEDS/HSD only (*n* = 167)	hEDS/HSD & Fibro (*n* = 414)	*P*-value*^a^*
**Patient sex**					
Female	42 (77.8%)	89 (90.8%)	146 (87.4%)	387 (93.5%)	0.002
Male	12 (22.2–%)	9 (9.2%)	21 (12.6%)	18 (4.3%)	<0.001
Non-binary	0 (0.0%)	0 (0.0%)	0 (0.0%)	9 (2.2%)	0.10
Other	0 (0.0%)	0 (0.0%)	0 (0.0%)	0 (0.0%)	
***Median age (range)***	36.7 (18.0, 70.0)	39.7 (18.0, 75.0)	35.0 (18.0, 71.0)	31.0 (18.0, 63.0)	<0.001
**Race**					
American Indian/Alaska native	0 (0.0%)	1 (1.0%)	2 (1.2%)	10 (2.4%)	0.72
Asian	2 (3.7%)	0 (0.0%)	1 (0.6%)	8 (1.9%)	0.20
Black or African American	1 (1.9%)	3 (3.1%)	3 (1.8%)	17 (4.1%)	0.59
Native Hawaii/Pacific Islander	0 (0.0%)	0 (0.0%)	0 (0.0%)	0 (0.0%)	-
White	49 (90.7%)	92 (93.9%)	160 (95.8%)	391 (94.4%)	0.52
Other	0 (0.0%)	2 (2.0%)	3 (1.8%)	13 (3.1%)	0.64
Unknown/not disclosed	2 (3.7%)	1 (1.0%)	2 (1.2%)	3 (0.7%)	0.20
***Ethnicity***					0.06
Hispanic/Latino	2 (3.7%)	9 (9.2%)	13 (7.8%)	29 (7.0%)	
Not Hispanic/Latino	47 (87.0%)	84 (85.7%)	151 (90.4%)	377 (91.1%)	
Choose not to disclose/unknown	5 (9.3%)	5 (5.1%)	3 (1.8%)	8 (1.9%)	
***Highest level of education***					0.14
Some high school	1 (1.9%)	1 (1.0%)	3 (1.8%)	4 (1.0%)	
High school/GED	4 (7.4%)	9 (9.2%)	9 (5.4%)	32 (7.7%)	
Some college	7 (13.0%)	26 (26.5%)	38 (22.8%)	107 (25.8%)	
Trade school	2 (3.7%)	3 (3.1%)	4 (2.4%)	27 (6.5%)	
Associates	8 (14.8%)	8 (8.2%)	21 (12.6%)	48 (11.6%)	
Bachelors	21 (38.9%)	25 (25.5%)	41 (24.6%)	119 (28.7%)	
Masters	6 (11.1%)	22 (22.4%)	34 (20.4%)	51 (12.3%)	
Professional/doctorate	5 (9.3%)	4 (4.1%)	16 (9.6%)	24 (5.8%)	
Not disclosed	0 (0.0%)	0 (0.0%)	1 (0.6%)	2 (0.5%)	
***Cigarette smoke exposure***					0.59
Yes	21 (38.9%)	48 (49.0%)	66 (39.5%)	189 (45.7%)	
No	33 (61.1%)	50 (51.0%)	99 (59.3%)	220 (53.1%)	
Unknown	0 (0.0%)	0 (0.0%)	2 (1.2%)	5 (1.2%)	

^a^*P*-values result from Fisher’s test for categorical data and Kruskal–Wallis rank sum test for numeric data.

### Patient history of hEDS/HSD

We describe the data for patients that answered “yes” in the questionnaire for all four groups in Table 2. A table that includes all data in men and women such as missing data and those who answered “yes,” “no,” or “unknown” to questions is found in Supplementary Table 1. We examined whether patients had been evaluated for hEDS or HSD prior to coming to the EDS Clinic in Table 2 and found that 61.6% of patients with hEDS/HSD&Fibro had been previously evaluated compared to controls (*p* < 0.001) and 36.5% given a diagnosis of hEDS (*p* < 0.001). When we asked if a family member had a similar diagnosis, we found that 43.9% of Fibro (*p* = 0.03), 56.3% of hEDS/HSD (*p* = 0.004), and 66.7% of hEDS/HSD&Fibro (*p* < 0.001) patients answered yes compared to controls (Table 2). Because hEDS/HSD occurs primarily in women, we also analyzed the conditions/symptoms for the four groups of patients in women only (Supplementary Table 2). Almost identical results were found with 61.2% of women with hEDS/HSD&Fibro being previously evaluated for a hypermobility syndrome compared to controls (*p* < 0.001) and 36.4% given a diagnosis of hEDS (*p* < 0.001). When we asked if a family member had a similar diagnosis, we found that 43.8% of Fibro (*p* = 0.12), 56.8% of hEDS/HSD (*p* = 0.04), and 66.9% of hEDS/HSD&Fibro (*p* < 0.001) women answered yes compared to controls (Supplementary Table 2).

**TABLE 2:** Past history of hEDS/HSD in female and male patients diagnosed with fibromyalgia, hypermobility (hEDS or HSD), or hEDS/HSD and fibromyalgia compared to controls (*n* = 733).

Past history of hEDS/HSD	Control (no fibro,*^a^* hEDS/HSD) (*n* = 54)	Fibro only (*n* = 98)	hEDS/HSD (*n* = 167)	hEDS/HSD & Fibro (*n* = 414)	*P*-value*^b^*
Past hypermobile assessment?	16 (29.6%)	40 (40.8%)	71 (42.5%)	255 (61.6%)***	<0.001
Previously diagnosed with hEDS	4 (7.4%)	14 (14.3%)	29 (17.4%)	151 (36.5%)***	<0.001
Previously diagnosed with HSD	3 (5.6%)	5 (5.1%)	7 (4.2%)	24 (5.8%)	0.89
Previously diagnosed with general EDS?	1 (1.9%)	9 (9.2%)	5 (3.0%)	21 (5.1%)	0.14
Previously diagnosed with other type of EDS?	0 (0.0%)	5 (5.1%)	3 (1.8%)	12 (2.9%)	0.31
No previous EDS diagnosis	4 (7.4%)	4 (4.1%)	13 (7.8%)	32 (7.7%)	0.64
Family member with similar diagnosis	18 (33.3%)	43 (43.9%)*	94 (56.3%)**	276 (66.7%)***	<0.001

^a^Fibro, fibromyalgia; hEDS, hypermobile Ehlers-Danlos syndrome; HSD, hypermobility spectrum disorders. ^b^*P*-values result from Fisher’s test for categorical data and Kruskal-Wallis rank sum test for numeric data. *Post hoc* analysis compares Fibro, hEDS/HSD or hEDS/HSD&Fibro to control patients that are not diagnosed with hypermobility or fibromyalgia: *p < 0.05; **p < 0.01; ***p < 0.001.

### Joint issues, bruising, and scarring

When we evaluated patients for joint-related symptoms, we found that 59.9% of hEDS/HSD (*p* = 0.04), 76.5% of Fibro (*p* < 0.001) and 86.2% of hEDS/HSD&Fibro (*p* < 0.001) patients reported being clumsy compared to controls (Table 3). Joint pain was significantly increased in all groups compared to controls with 83.2% of hEDS/HSD (*p* = 0.03), 96.9% of Fibro (*p* < 0.001), and 97.3% of hEDS/HSD&Fibro (*p* < 0.001) patients affected (Table 3). Joint pain was worse after menses or after writing and typing only for patients with Fibro (*p* < 0.001, *p* = 0.01, respectively) or hEDS/HSD&Fibro (*p* < 0.001, *p* < 0.001, respectively) (Table 3). Similarly, jaw pain (jaw clicking/temporomandibular joint dysfunction- TMJ) was only significantly increased over controls in patients with Fibro (*p* = 0.04) or hEDS/HSD&Fibro (*p* < 0.001). We found that subluxations were significantly increased in all three groups compared to controls with 64.3% of Fibro (*p* = 0.02), 65.9% of hEDS/HSD (*p* = 0.006) and 81.2% of hEDS/HSD&Fibro (*p* < 0.001) patients self-reporting this problem (Table 3). However, only hEDS/HSD&Fibro patients self-reported significantly more joint issues like sprains (*p* < 0.001) and dislocations (*p* = 0.01). Interestingly, 20.4% of controls reported no joint issues compared to the hypermobile and fibromyalgia groups where few patients had no joint issues (1–4%) (*p* < 0.001). Patients with Fibro or hEDS/HSD&Fibro reported that joint pain kept them from daily activities (88.7% *p* < 0.001, 89% *p* < 0.001, respectively) and were worse after they stopped sports (55.2% *p* = 0.003, 64.6% *p* < 0.001, respectively) (Table 3). Significantly more hEDS/HSD&Fibro patients stopped sports because of joint injuries (*p* = 0.006) compared to controls, which was not significant for other groups. More patients in this group also reported having scoliosis (*p* = 0.04). The main difference in men and women (Table 3) vs. women only (Supplementary Table 2) is that joint issues were only significantly increased compared to controls in women with Fibro or hEDS/HSD&Fibro, but not for patients with only hEDS/HSD where symptoms and comorbidities occurred significantly less often than in patients with Fibro ± hEDS/HSD.

**TABLE 3:** Joint issues, bruising, and scarring in female and male patients diagnosed with fibromyalgia, hypermobility (hEDS or HSD) or hEDS/HSD and fibromyalgia compared to controls (*n* = 733).

Joint issues, bruising and scarring	Control (no fibro*^a^*, hEDS/HSD) (*n* = 54)	Fibro only (*n* = 98)	hEDS/HSD (*n* = 167)	hEDS/HSD & Fibro (*n* = 414)	*P*-value*^b^*
Clumsy	24 (44.4%)	75 (76.5%)***	100 (59.9%)*	357 (86.2%)***	<0.001
Joint pain	37 (68.5%)	95 (96.9%)***	139 (83.2%)*	403 (97.3%)***	<0.001
Joint pain worse during menses?	8 (19.0%)	50 (56.2%)***	46 (31.5%)	212 (54.8%)***	<0.001
Do you have hand pain after writing or typing?	31 (72.1%)	87 (89.7%)*	114 (71.2%)	374 (91.4%)***	<0.001
Jaw clicks/TMJ	35 (64.8%)	74 (75.5%)*	103 (61.7%)	363 (87.7%)***	<0.001
Joint issues, e.g., sprains	24 (44.4%)	59 (60.2%)	97 (58.1%)	325 (78.5%)***	<0.001
Subluxations	24 (44.4%)	63 (64.3%)*	110 (65.9%)**	336 (81.2%)***	<0.001
Dislocations	13 (24.1%)	25 (25.5%)	42 (25.1%)	153 (37.0%)	0.01
No joint issues	11 (20.4%)	1 (1.0%)***	7 (4.2%)***	5 (1.2%)***	<0.001
Were you involved in sports that required flexibility (e.g., gymnastics, dance)	26 (48.1%)	48 (49.0%)	92 (55.1%)	260 (62.8%)	0.045
Stop sports due to injury?	22 (52.4%)	26 (38.8%)	76 (56.7%)	251 (74.0%)**	<0.001
Worse symptoms after stopping sports?	14 (33.3%)	37 (55.2%)**	56 (41.8%)	219 (64.6%)***	<0.001
Does joint pain keep you from daily activities?	26 (60.5%)	86 (88.7%)***	94 (58.8%)	364 (89.0%)***	<0.001
Scoliosis	17 (31.5%)	40 (40.8%)	43 (25.7%)	153 (37.0%)*	0.002
Have you ever broken a bone?	31 (57.4%)	61 (62.2%)	83 (49.7%)	255 (61.6%)	0.045
History of significant surgeries on joints, bones or ligaments?	17 (31.5%)	36 (36.7%)	52 (31.1%)	160 (38.6%)	0.64
Easily bruised	28 (51.9%)	80 (81.6%)***	122 (73.1%)**	353 (85.3%)***	<0.001
History of easy scarring	21 (38.9%)	67 (68.4%)***	92 (55.1%)*	321 (77.5%)***	<0.001
Poor wound healing	19 (35.2%)	54 (55.1%)*	58 (34.7%)	288 (69.6%)***	<0.001

^a^Fibro, fibromyalgia; hEDS, hypermobile Ehlers-Danlos syndrome; HSD, hypermobility spectrum disorders; TMJ, temporomandibular joint dysfunction. ^b^*P*-values result from Fisher’s test for categorical data and Kruskal–Wallis rank sum test for numeric data. Post hoc analysis compares Fibro, hEDS/HSD or hEDS/HSD&Fibro to control patients that were not diagnosed with hypermobility or fibromyalgia: *p < 0.05; **p < 0.01; ***p < 0.001.

All three groups reported being easily bruised and a history of easy scarring that was significantly increased over controls (*p* < 0.001), while significantly more Fibro and hEDS/HSD&Fibro patients reported having poor wound healing compared to controls (*p* < 0.001) (Table 3). Similar results were observed for females (Supplementary Table 2).

### Allergy/atopy and hay fever

Mast cells are known to play a key role in the etiology or exacerbation of headaches and migraine (22, 23). Mast cells are also central to remodeling (break down and build-up of the extracellular matrix) and fibrosis (collagen deposition leading to scar) by releasing many enzymes such as Serpin A3n (α1-antichymotrypsin) which activates cytokines like interleukin (IL)-1β and matrix metalloproteinases (MMPs) that are essential in the remodeling and fibrosis process (24–26). Resident mast cells are located in all tissues and interstitial sites and respond to tissue damage with a central role in recruiting inflammation to the site of damage where they can act as antigen presenting cells expressing MHC class II, as well as releasing proinflammatory mediators like cytokines that also recruit other immune cells to the site (24, 25, 27). Other comorbidities that are commonly reported by patients with hEDS/HSD and fibromyalgia such as dyspepsia and irritable bowel syndrome (IBS) have been linked to mast cell activation/hyperactivity (28, 29). For these reasons we examined whether patients self-reported mast cell activation in the form of allergy, atopy or hayfever. We found that allergy/atopy occurred in a large percentage of patients in all three groups (around 70–80% of patients) but was also high in controls (66%) (Table 4). However, only patients with hEDS/HSD&Fibro reported significantly more allergies over controls (*p* = 0.004). Hayfever was self-reported in more Fibro patients than controls (*p* = 0.04) but was not more frequent in other groups. In contrast, allergies/atopy and hayfever did not occur more often in any of the three groups compared to controls when only women were examined (pair-wise comparisons, Supplementary Table 2).

**TABLE 4:** Allergy, headaches, migraine, and related conditions in female and male patients diagnosed with fibromyalgia, hypermobility (hEDS or HSD) or hEDS/HSD and fibromyalgia compared to controls (*n* = 733).

Allergy or neurologic condition	Control (no fibro*^a^*, hEDS/HSD) (*n* = 54)	Fibro only (*n* = 98)	hEDS/HSD (*n* = 167)	hEDS/HSD & Fibro (*n* = 414)	*P*-value*^b^*
Allergy/atopy	31 (66.0%)	68 (77.3%)	106 (69.7%)	303 (81.5%)**	0.002
Hayfever	19 (35.2%)	52 (53.1%)*	52 (31.1%)	186 (44.9%)	<0.001
Headache	19 (35.2%)	74 (75.5%)***	101 (60.5%)**	335 (80.9%)***	<0.001
Daily persistent headache	4 (7.4%)	32 (32.7%)***	27 (16.2%)	158 (38.2%)***	<0.001
Cluster headache	5 (9.3%)	10 (10.2%)	6 (3.6%)	71 (17.1%)	<0.001
Migraine	20 (37.0%)	42 (42.9%)	71 (42.5%)	264 (63.8%)***	<0.001
Chronic migraine	10 (18.5%)	29 (29.6%)	23 (13.8%)	141 (34.1%)*	<0.001
Chiari malformation	1 (1.9%)	8 (8.2%)	0 (0.0%)	27 (6.5%)	<0.001
Intracranial hypertension	1 (1.9%)	5 (5.1%)	2 (1.2%)	28 (6.8%)	0.018
CSF leak	1 (1.9%)	3 (3.1%)	1 (0.6%)	18 (4.3%)	0.087
Current or past abnormal brain MRI?	1 (1.9%)	17 (17.3%)**	8 (4.8%)	59 (14.3%)**	<0.001
Autonomic dysfunction	6 (11.1%)	39 (39.8%)***	18 (10.8%)	157 (37.9%)***	<0.001
Neuropathy	7 (13.0%)	44 (44.9%)***	28 (16.8%)	134 (32.4%)**	<0.001
Brain fog	22 (40.7%)	87 (88.8%)***	82 (49.1%)	369 (89.1%)***	<0.001
Vertigo	11 (20.4%)	38 (38.8%)*	31 (18.6%)	189 (45.7%)***	<0.001
Tinnitus	12 (22.2%)	57 (58.2%)***	49 (29.3%)	227 (54.8%)***	<0.001

^a^CSF, cerebral spinal fluid; Fibro, fibromyalgia; hEDS, hypermobile Ehlers-Danlos syndrome; HSD, hypermobility spectrum disorders; MRI, magnetic resonance imaging. ^b^*P*-values result from Fisher’s test for categorical data and Kruskal–Wallis rank sum test for numeric data. Post hoc analysis compares Fibro, hEDS/HSD or hEDS/HSD&Fibro to control patients that were not diagnosed with hypermobility or fibromyalgia: *p < 0.05; **p < 0.01; ***p < 0.001.

### Headaches, migraine, and related issues

Headache was reported to occur significantly more often in all three groups with 60.5% of hEDS/HSD (*p* = 0.002), 75.5% of Fibro (*p* < 0.001), and 80.9% of hEDS/HSD&Fibro (*p* < 0.001) patients with symptoms compared to controls (Table 4). Daily persistent headache, which has been associated with hEDS (30), occurred in significantly more patients with Fibro (32.7%, *p* < 0.001) and hEDS/HSD&Fibro (38.2%, *p* < 0.001) than hEDS/HSD only patients (16.2%, *p* = 0.12) compared to controls. Migraine (63.8% vs. 37% in controls and 43% for Fibro and hEDS/HSD) and chronic migraine were only significantly higher than controls in hEDS/HSD&Fibro patients (*p* < 0.001 and *p* = 0.02, respectively). A past or current abnormal MRI was also self-reported more often in patients with Fibro or hEDS/HSD&Fibro rather than hEDS/HSD patients (Table 4). The same results were found for women (Supplementary Table 2).

We found that autonomic dysfunction, neuropathy, brain fog, vertigo, and tinnitus were self-reported in significantly more patients with Fibro or hEDS/HSD&Fibro compared to controls and hEDS/HSD patients (Table 4). Brain fog occurred in around 90% of patients with Fibro or hEDS/HSD&Fibro, but only occurred in 49% of hEDS/HSD patients (*p* < 0.001). The same results and very similar percentages were reported for women (Supplementary Table 2).

### Stomach and intestinal issues

Nausea, heartburn/dyspepsia, vomiting, diarrhea, constipation, and IBS were all reported significantly more often in patients with Fibro or hEDS/HSD&Fibro compared to controls or hEDS/HSD patients (Table 5). The most frequent symptom in this category was nausea which occurred in 70.4% of Fibro (*p* < 0.001) and 76.6% of hEDS/HSD&Fibro (*p* < 0.001) patients compared to around 30% of controls and hEDS/HSD patients (Table 5). Also frequent was constipation, which occurred in 64.3% of Fibro (*p* < 0.001) and 63.8% of hEDS/HSD&Fibro (*p* < 0.001) patients compared to around 30–40% of controls and hEDS/HSD patients. Diarrhea occurred in 57.1% of Fibro (*p* < 0.001) and 62.3% of hEDS/HSD&Fibro (*p* < 0.001) patients compared to around 20–30% of controls and hEDS/HSD patients (Table 5). In general, stomach and intestinal symptoms were self-reported in around 2× as many patients with Fibro compared to patients with hEDS/HSD without Fibro (Table 5; Supplementary Table 2).

**TABLE 5:** Stomach and intestinal issues in female and male patients diagnosed with fibromyalgia, hypermobility (hEDS or HSD) or hEDS/HSD and fibromyalgia compared to controls (*n* = 733).

Stomach and intestinal issues	Control (no fibro*^a^*, hEDS/HSD) (*n* = 54)	Fibro only (*n* = 98)	hEDS/HSD (*n* = 167)	hEDS/HSD & Fibro (*n* = 414)	*P*-value*^b^*
Nausea	16 (29.6%)	69 (70.4%)***	51 (30.5%)	317 (76.6%)***	<0.001
Heartburn	8 (14.8%)	39 (39.8%)**	28 (16.8%)	169 (40.8%)***	<0.001
Vomiting	9 (16.7%)	31 (31.6%)	17 (10.2%)	179 (43.2%)***	<0.001
Diarrhea	12 (22.2%)	56 (57.1%)***	56 (33.5%)	258 (62.3%)***	<0.001
Constipation	17 (31.5%)	63 (64.3%)***	72 (43.1%)	264 (63.8%)***	<0.001
IBS	10 (18.5%)	46 (46.9%)***	44 (26.3%)	172 (41.5%)***	<0.001
Crohn’s disease	2 (3.7%)	1 (1.0%)	0 (0.0%)	11 (2.7%)	0.07
Ulcerative colitis	3 (5.6%)	5 (5.1%)	0 (0.0%)*	8 (1.9%)	0.006

^a^Fibro, fibromyalgia; hEDS, hypermobile Ehlers-Danlos syndrome; HSD, hypermobility spectrum disorders; IBS, irritable bowel syndrome. ^b^*P*-values result from Fisher’s test for categorical data and Kruskal–Wallis rank sum test for numeric data. Post hoc analysis compares Fibro, hEDS/HSD or hEDS/HSD&Fibro to control patients that were not diagnosed with hypermobility or fibromyalgia: *p < 0.05; **p < 0.01; ***p < 0.001.

### Mental health issues and abuse

Recent studies found co-occurrence of patients and symptoms/comorbidities with hEDS/HSD and autism/autism spectrum disorder (ASD) (31–33). Patients in this study did not self-report more autism/ASD compared to controls for any group (*p* = 0.27, *p* = 0.36) (Table 6; Supplementary Table 2). All groups reported a high percentage of anxiety, but only patients with hEDS/HSD&Fibro (75.1%, *p* < 0.001) reported significantly more anxiety than controls (51.9%) (Table 6), which was also true for women (74.9% vs. 59.9%) (Supplementary Table 2). Depression occurred more often in patients with Fibro (59.2%, *p* < 0.001) and hEDS/HSD&Fibro (60.1%, *p* < 0.001) which occurred in around 30–35% of controls and hEDS/HSD patients (Table 6). Around 60% of women with Fibro or hEDS/HSD&Fibro also reported depression (Supplementary Table 2).

**TABLE 6:** Mental health issues and abuse in female and male patients diagnosed with fibromyalgia, hypermobility (hEDS or HSD) or hEDS/HSD and fibromyalgia compared to controls (*n* = 733).

Condition/symptom	Control (no fibro*^a^*, hEDS/HSD) (*n* = 54)	Fibro only (*n* = 98)	hEDS/HSD (*n* = 167)	hEDS/HSD & Fibro (*n* = 414)	*P*-value*^b^*
Autism/ASD	1 (1.9%)	5 (5.1%)	4 (2.4%)	24 (5.8%)	0.273
Anxiety	28 (51.9%)	65 (66.3%)	82 (49.1%)	311 (75.1%)***	<0.001
Depression	16 (29.6%)	58 (59.2%)***	61 (36.5%)	249 (60.1%)***	<0.001
PTSD	3 (5.6%)	31 (31.6%)***	18 (10.8%)	136 (32.9%)***	<0.001
Have you ever been abused?	10 (18.5%)	34 (34.7%)	28 (16.8%)	148 (35.7%)**	<0.001
Emotional abuse	9 (16.7%)	28 (28.6%)	20 (12.0%)	123 (29.7%)	<0.001
Physical abuse	7 (13.0%)	18 (18.4%)	10 (6.0%)	80 (19.3%)	<0.001
Sexual abuse	3 (5.6%)	22 (22.4%)**	13 (7.8%)	93 (22.5%)**	<0.001
Unknown/chose not to disclose abuse	0 (0.0%)	4 (4.1%)	3 (1.8%)	4 (1.0%)	0.129

^a^ASD, autism spectrum disorder; Fibro, fibromyalgia; hEDS, hypermobile Ehlers-Danlos syndrome; HSD, hypermobility spectrum disorders; PTSD, post-traumatic stress disorder. ^b^*P*-values result from Fisher’s test for categorical data and Kruskal–Wallis rank sum test for numeric data. Post hoc analysis compares Fibro, hEDS/HSD or hEDS/HSD&Fibro to control patients that were not diagnosed with hypermobility or fibromyalgia: **p < 0.01; ***p < 0.001.

Around 33% of Fibro and hEDS/HSD&Fibro patients reported PTSD (*p* < 0.001) (Table 6), which was the same for women (Supplementary Table 2), and slightly higher levels reported having been abused (around 35% for Fibro conditions compared to 17% in hEDS/HSD patients). Sexual abuse was reported in around 22% of patients with Fibro or hEDS/HSD&Fibro (*p* < 0.001), but only around 6–8% of controls and hEDS/HSD patients (Table 6; Supplementary Table 2).

### Overlap between symptoms/conditions

**FIGURE 1:**
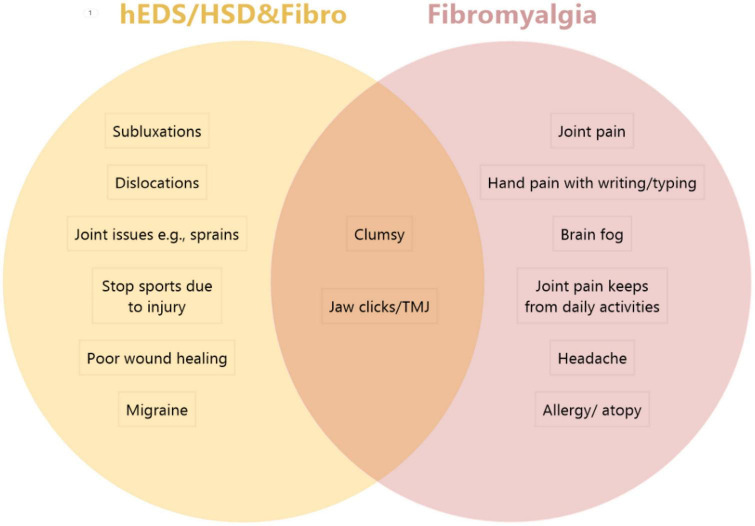
Comparison of top symptoms/comorbidities in patients with hEDS/HSD&Fibro vs. Fibro. The top 5 issues that were significantly worse in hEDS/HSD and fibromyalgia patients (hEDS/HSD&Fibro) from Table 7 included (in order) subluxations, joint issues (e.g., sprains), stop sports due to injury, poor wound healing and migraine. The top issues in Fibromyalgia patients from Supplementary Table 3 in order included joint pain, hand pain with writing/typing, brain fog, joint pain keeps from daily activities, headache and allergy/atopy. Patients with hEDS&Fibro also reported dislocations as a top issue that was significantly different in Table 10. Top issues in both patient groups included being clumsy and jaw clicks/TMJ.

To determine whether significant overlap occurred between the symptoms/conditions reported between groups we conducted pair-wise statistical comparisons of Fibro to hEDS/HSD&Fibro or hEDS/HSD to hEDS/HSD&Fibro (Table 7). The full 40 conditions are listed from most frequently self-reported to least reported for the hEDS/HSD&Fibro group in Table 7, for Fibro in Supplementary Table 3 and for hEDS/HSD in Supplementary Table 4 and summarized for the top 15 issues for each group in Table 8. There were no significant differences in 33 conditions listed in Table 7 between Fibro vs. hEDS/HSD&Fibro patients indicating that those issues were related to fibromyalgia rather than hEDS/HSD. The only issues that were significantly different between Fibro and hEDS/HSD&Fibro patients indicating that they were associated with the co-occurrence of hEDS/HSD with fibromyalgia were subluxations, being clumsy, joint issues such as sprains, stopping sports due to injury, jaw clicks/TMJ, poor wound healing and migraine (bold in Table 7; Figure 1). Importantly, hEDS/HSD patients without fibromyalgia were significantly different from hEDS/HSD&Fibro patients for all 40 conditions (Table 7) indicating that the conditions occurred far more often in hEDS/HSD&Fibro patients than hEDS/HSD patients. The same results were obtained for women, where 5 out of 40 issues were significantly associated with co-occurrence of hEDS/HSD&Fibro including subluxations, joint issues such as sprains, stopping sports due to injury, poor wound healing, and migraine while 3 additional symptoms including jaw clicks/TMJ, being clumsy and worse symptoms after stopping sports were borderline significant (bold in Supplementary Table 5).

**TABLE 7:** Comparison of hEDS/HSD&Fibro (*n* = 414) to Fibro (*n* = 98) or hEDS/HSD (*n* = 167) in men and women.

Condition*^a^*	hEDS/HSD & Fibro	Fibro	*P*-value*^b^*	hEDS/HSD	*P*-value*^c^*
Joint pain	97.3%	96.9%	*p* > 0.99	83.2%	*p* < 0.001
Hand pain with writing/typing	91.4%	89.7%	*p* > 0.99	71.2%	*p* < 0.001
Brain fog	89.1%	88.8%	*p* > 0.99	49.1%	*p* < 0.001
Joint pain keeps from daily activities	89.0%	88.7%	*p* > 0.99	58.8%	*p* < 0.001
**Jaw clicks, TMJ*^d^***	87.7%	75.5%	*p* = 0.01	61.7%	*p* < 0.001
Clumsy	86.2%	76.5%	*p* = 0.04	59.9%	*p* < 0.001
Allergy/Atopy	81.5%	77.3%	*p* > 0.99	69.7%	*p* = 0.001
**Subluxations**	81.2%	64.3%	*p* = 0.001	65.9%	*p* < 0.001
Headache	80.9%	75.5%	*p* = 0.52	60.5%	*p* < 0.001
Joint issues, e.g., sprains	78.5%	60.2%	*p* < 0.001	58.1%	*p* < 0.001
History of easy scarring	77.5%	68.4%	*p* = 0.13	55.1%	*p* < 0.001
Nausea	76.6%	70.4%	*p* = 0.48	30.5%	*p* < 0.001
Anxiety	75.1%	66.3%	*p* = 0.20	49.1%	*p* < 0.001
**Stop sports due to injury**	74.0%	38.8%	*p* < 0.001	56.7%	*p* < 0.001
**Poor wound healing**	69.6%	55.1%	*p* = 0.02	34.7%	*p* < 0.001
Worse joint problems after stopping sports	64.6%	55.2%	*p* = 0.16	41.8%	*p* < 0.001
Constipation	63.8%	64.3%	*p* > 0.99	43.1%	*p* < 0.001
**Migraine**	63.8%	42.9%	*p* < 0.001	42.5%	*p* < 0.001
Diarrhea	62.3%	57.1%	*p* = 0.72	33.5%	*p* < 0.001
Have you ever broken a bone?	61.6%	62.2%	*p* = 0.21	49.7%	*p* = 0.03
Depression	60.1%	59.2%	*p* > 0.99	36.5%	*p* < 0.001
Joint pain with menses	54.8%	56.2%	*p* > 0.99	31.5%	*p* < 0.001
Tinnitus	54.8%	58.2%	*p* > 0.99	29.3%	*p* < 0.001
Vertigo	45.7%	38.8%	*p* = 0.52	18.6%	*p* < 0.001
Hayfever	44.9%	53.1%	*p* = 0.35	31.1%	*p* = 0.006
Vomiting	43.2%	31.6%	*p* = 0.08	10.2%	*p* < 0.001
Irritable bowel syndrome (IBS)	41.5%	46.9%	*p* = 0.25	26.3%	*p* = 0.02
Heartburn	40.8%	39.8%	*p* > 0.99	16.8%	*p* < 0.001
Daily persistent headache	38.2%	32.7%	*p* = 0.71	16.2%	*p* < 0.001
Autonomic dysfunction	37.9%	39.8%	*p* > 0.99	10.8%	*p* < 0.001
Dislocations	37.0%	25.5%	*p* = 0.07	25.1%	*p* = 0.01
Scoliosis	37.0%	40.8%	*p* > 0.99	25.7%	*p* < 0.001
History of abuse	35.7%	34.7%	*p* > 0.99	16.8%	*p* < 0.001
Chronic migraine (> 8 days/month)	34.1%	29.6%	*P* = 0.95	13.8%	*p* < 0.001
PTSD	32.9%	31.6%	*p* > 0.99	10.8%	*p* < 0.001
Neuropathy	32.4%	44.9%	*p* = 0.03	16.8%	*p* = 0.004
Emotional abuse	29.7%	28.6%	*p* > 0.99	12.0%	*p* < 0.001
Sexual abuse	22.5%	22.4%	*p* > 0.99	7.8%	*p* < 0.001
Physical abuse	19.3%	18.4%	*p* > 0.99	6.0%	*p* < 0.001

^a^Order of conditions based on highest to lowest% in patients diagnosed with hEDS/HSD&Fibro. ^b^Adjusted *P*-values compare Fibro control to hEDS/HSD&Fibro. ^c^Adjusted *P*-values compare hEDS/HSD to hEDS/HSD&Fibro. ^d^Conditions primarily associated with hEDS/HSD and Fibro are marked in *bold*. All other conditions occur more often in patients with Fibro (not bold).

**TABLE 8:** Top 15 self-reported issues in patients with hEDS/HSD, Fibro or hEDS/HSD&Fibro in men and women.

Top 15 issues in hEDS/HSD	Top 15 issues in Fibromyalgia	Top 15 issues in hEDS/HSD&Fibro
Joint pain	Joint pain	Joint pain
Hand pain with writing/typing	Hand pain with writing/typing	Hand pain with writing/typing
Subluxations	Brain fog	Brain fog
Jaw clicks/TMJ	Joint pain keeps from daily activities	Joint pain keeps from daily activities
Headache	Clumsy	Jaw clicks/TMJ
Clumsy	Jaw clicks/TMJ	Clumsy
Joint pain keeps from daily activities	Allergy/atopy	Easy bruising
Joint issues, e.g., sprains	Headache	Allergy/atopy
Stop sports due to injury	Nausea	Subluxations
History of easy scarring	History of easy scarring	Headache
Brain fog	Anxiety	Joint issues, e.g., sprains
Anxiety	Constipation	History of easy scarring
Constipation	Subluxations	Nausea
Migraine	Joint issues, e.g., sprains	Anxiety
Worse joint problems after stopping	Depression	Stop sports due to injury

We also examined whether there were any differences in these parameters if the patient had hEDS or HSD and fibromyalgia. We found the same results as shown in Table 7 for patients with HSD&Fibro (Table 9) and patients with hEDS&Fibro (Table 10) except that hEDS&Fibro patients also had more dislocations. Thus, patients with a diagnosis of fibromyalgia and a hypermobile syndrome had worse symptoms/comorbidities regardless of whether they were diagnosed with HSD or hEDS. However, far more patients going to the EDS Clinic were diagnosed with HSD (76.6%) than hEDS (23.4%), so most patients had HSD&Fibro rather than hEDS&Fibro. Thus, more patients with fibromyalgia, regardless of whether they had HSD or hEDS, had all 40 of the symptoms/comorbidities listed in Table 7, whereas worse disease in patients with hEDS/HSD&Fibro was significantly associated with 7 of these issues of which 2 overlapped with the top fibromyalgia issues so that 5 issues distinguished patients with hEDS/HSD&Fibro from those with fibromyalgia (Table 7; Figure 1). Thus, our data show that patients with fibromyalgia and HSD or hEDS have worse disease based on more symptoms and comorbidities.

**TABLE 9:** Comparison of HSD&Fibro (*n* = 327) to Fibro (*n* = 98) or HSD (*n* = 128) in men and women.

Condition*^a^*	HSD & Fibro	Fibro	*P*-value*^b^*	HSD	*P*-value*^c^*
Joint pain	97.2%	96.9%	*p* > 0.99	84.4%	*p* < 0.001
Hand pain with writing/typing	90.4%	89.7%	*p* > 0.99	70.2%	*p* < 0.001
Brain fog	90.5%	88.8%	*p* > 0.99	52.3%	*p* < 0.001
Joint pain keeps from daily activities	87.9%	88.7%	*p* > 0.99	57.0%	*p* < 0.001
**Jaw clicks, TMJ*^d^***	87.5%	75.5%	*p* = 0.03	61.7%	*p* < 0.001
Clumsy	85.9%	76.5%	*p* = 0.047	59.4%	*p* < 0.001
Allergy/Atopy	81.9%	77.3%	*p* > 0.99	71.2%	*p* = 0.003
**Subluxations**	80.1%	64.3%	*p* = 0.003	65.6%	*p* < 0.001
Headache	80.1%	75.5%	*p* = 0.65	57.0%	*p* < 0.001
**Joint issues, e.g., sprains**	78.0%	60.2%	*p* = 0.001	57.8%	*p* < 0.001
History of easy scarring	77.1%	68.4%	*p* = 0.17	55.5%	*p* < 0.001
Nausea	77.7%	70.4%	*p* = 0.28	31.2%	*p* < 0.001
*Anxiety*	77.7%	66.3%	*p* = 0.07	49.2%	*p* < 0.001
**Stop sports due to injury**	73.1%	38.8%	*p* < 0.001	54.9%	*p* < 0.001
*Poor wound healing*	67.6%	55.1%	*p* = 0.06	32.8%	*p* < 0.001
Worse joint problems after stopping sports	63.4%	55.2%	*p* = 0.18	41.2%	*p* < 0.001
Constipation	64.5%	64.3%	*p* > 0.99	42.2%	*p* < 0.001
**Migraine**	63.9%	42.9%	*p* < 0.001	42.2%	*p* < 0.001
Diarrhea	62.7%	57.1%	*p* = 0.69	33.6%	*p* < 0.001
Have you ever broken a bone?	61.8%	62.2%	*p* = 0.13	47.7%	*p* = 0.041
Depression	60.6%	59.2%	*p* > 0.99	35.9%	*p* < 0.001
Joint pain with menses	54.8%	56.2%	*p* > 0.99	33.6%	*p* < 0.001
Tinnitus	54.4%	58.2%	*p* > 0.99	29.7%	*p* < 0.001
Vertigo	47.1%	38.8%	*p* = 0.33	21.9%	*p* < 0.001
Hayfever	45.0%	53.1%	*p* = 0.34	31.2%	*p* = 0.011
*Vomiting*	43.7%	31.6%	*p* = 0.07	10.9%	*p* < 0.001
Irritable bowel syndrome (IBS)	39.8%	46.9%	*p* = 0.49	28.1%	*p* < 0.001
Heartburn	43.4%	39.8%	*p* > 0.99	15.6%	*p* < 0.001
Daily persistent headache	39.8%	32.7%	*p* = 0.47	18.0%	*p* < 0.001
Autonomic dysfunction	35.8%	39.8%	*p* = 0.951	11.7%	*p* < 0.001
Dislocations	33.9%	25.5%	*p* = 0.28	25.0%	*p* = 0.003
Scoliosis	36.7%	40.8%	*p* = 0.65	26.6%	*p* = 0.001
History of abuse	36.7%	34.7%	*p* > 0.99	19.5%	*p* < 0.001
Chronic migraine (> 8 days/month)	34.9%	29.6%	*p* = 0.79	14.1%	*p* < 0.001
PTSD	35.8%	31.6%	*p* = 0.94	12.5%	*p* < 0.001
*Neuropathy*	32.7%	44.9%	*p* = 0.06	18.0%	*p* < 0.001
Emotional abuse	31.2%	28.6%	*p* > 0.99	14.8%	*p* < 0.001
Sexual abuse	22.0%	22.4%	*p* > 0.99	9.4%	*p* < 0.001
Physical abuse	20.5%	18.4%	*p* > 0.99	7.0%	*p* = 0.002

^a^Order of conditions based on highest to lowest % in patients diagnosed with hHSD&Fibro. ^b^Adjusted *P*-values compare Fibro to HSD&Fibro. ^c^Adjusted *P*-values compare HSD to HSD&Fibro. ^d^Conditions primarily associated with HSD and Fibro are marked in *bold* and conditions that were close to being significant are *italicized*. All other conditions occur more often in patients with Fibro (not bold).

**TABLE 10:** Comparison of hEDS&Fibro (*n* = 87) to Fibro (*n* = 98) or hEDS (*n* = 39) in men and women.

Condition*^a^*	hEDS & Fibro	Fibro	*P*-value*^b^*	hEDS	*P*-value*^c^*
Joint pain	97.7%	96.9%	*p* > 0.99	79.5%	*p* < 0.001
Hand pain with writing/typing	95.3%	89.7%	*p* = 0.35	74.4%	*p* < 0.001
Brain fog	83.9%	88.8%	*p* = 0.78	38.5%	*p* < 0.001
Joint pain keeps from daily activities	93.0%	88.7%	*p* = 0.89	64.1%	*p* < 0.001
**Jaw clicks, TMJ*^d^***	88.5%	75.5%	*p* = 0.03	61.5%	*p* < 0.001
Clumsy	87.4%	76.5%	*p* = 0.20	61.5%	*p* < 0.001
Allergy/Atopy	79.8%	77.3%	*p* > 0.99	64.7%	*p* = 0.003
**Subluxations**	85.1%	64.3%	*p* = 0.003	66.7%	*p* < 0.001
Headache	83.9%	75.5%	*p* = 0.40	71.8%	*p* < 0.001
**Joint issues, e.g., sprains**	80.5%	60.2%	*p* = 0.007	59.0%	*p* < 0.001
History of easy scarring	79.3%	68.4%	*p* = 0.20	53.8%	*p* < 0.001
Nausea	72.4%	70.4%	*p* > 0.99	28.2%	*p* < 0.001
Anxiety	65.5%	66.3%	*p* > 0.99	48.7%	*p* < 0.001
**Stop sports due to injury**	77.5%	38.8%	*p* < 0.001	62.5%	*p* < 0.001
**Poor wound healing**	77.0%	55.1%	*p* = 0.004	41.0%	*p* < 0.001
Worse joint problems after stopping sports	69.0%	55.2%	*p* = 0.16	43.8%	*p* < 0.001
Constipation	60.9%	64.3%	*p* > 0.99	46.2%	*p* < 0.001
**Migraine**	63.2%	42.9%	*p* = 0.02	43.6%	*p* < 0.001
Diarrhea	60.9%	57.1%	*p* > 0.99	33.3%	*p* < 0.001
Have you ever broken a bone?	60.9%	62.2%	*p* > 0.99	56.4%	*p* = 0.041
Depression	58.6%	59.2%	*p* > 0.99	38.5%	*p* < 0.001
Joint pain with menses	54.7%	56.2%	*p* = 0.72	22.2%	*p* < 0.001
Tinnitus	56.3%	58.2%	*p* > 0.99	28.2%	*p* < 0.001
Vertigo	40.2%	38.8%	*p* > 0.99	7.7%	*p* < 0.001
Hayfever	44.8%	53.1%	*p* = 0.61	30.8%	*p* = 0.011
Vomiting	41.4%	31.6%	*p* = 0.440	7.7%	*p* < 0.001
Irritable bowel syndrome (IBS)	48.3%	46.9%	*p* > 0.99	20.5%	*p* < 0.001
Heartburn	31.0%	39.8%	*p* = 0.45	20.5%	*p* < 0.001
Daily persistent headache	32.2%	32.7%	*p* > 0.99	10.3%	*p* < 0.001
Autonomic dysfunction	46.0%	39.8%	*p* = 0.91	7.7%	*p* < 0.001
**Dislocations**	48.3%	25.5%	*p* = 0.004	25.6%	*p* = 0.003
Scoliosis	37.9%	40.8%	*p* > 0.99	23.1%	*p* = 0.001
History of abuse	32.2%	34.7%	*p* > 0.99	7.7%	*p* < 0.001
Chronic migraine (> 8 days/month)	31.0%	29.6%	*p* > 0.99	12.8%	*p* < 0.001
PTSD	21.8%	31.6%	*p* = 0.28	5.1%	*p* < 0.001
Neuropathy	31.0%	44.9%	*p* = 0.14	12.8%	*p* < 0.001
Emotional abuse	24.1%	28.6%	*p* > 0.99	2.6%	*p* < 0.001
Sexual abuse	24.1%	22.4%	*p* > 0.99	2.6%	*p* < 0.001
Physical abuse	14.9%	18.4%	*p* > 0.99	2.6%	*p* = 0.002

^a^Order of conditions based on highest to lowest% in patients diagnosed with hEDS&Fibro. ^b^Adjusted *P*-values compare Fibro to hEDS&Fibro. ^c^Adjusted *P*-values compare hEDS to hEDS&Fibro. ^d^Conditions primarily associated with hEDS and Fibro are marked in *bold*. All other conditions occur more often in patients with Fibro (not bold).

## Discussion

A key finding of this study was that most (70%) patients seen in our EDS Clinic were diagnosed with fibromyalgia whereas around 23% were diagnosed with hEDS/HSD alone. Importantly, patients with the highest number of symptoms and comorbidities- the worst disease phenotype- had hEDS/HSD plus fibromyalgia (56% of patients), indicating that fibromyalgia and hEDS/HSD are not mutually exclusive and can co-exist together. Additionally, the high overlap in percent of patients with the same 40 symptoms and comorbidities that were diagnosed with both hEDS/HSD and Fibro suggest that hEDS/HSD may lead to fibromyalgia or be a secondary condition in patients that have additional environmental “hits” such as a serious infection, physical or psychological trauma. That hEDS/HSD is typically diagnosed at an earlier age than fibromyalgia is additional support of this idea (age 55 for fibromyalgia and an average age in 30s in our study, although over 60% had been assessed prior to coming to our EDS Clinic) (1). Fibromyalgia has been reported to occur as a secondary condition or co-occur in 15–30% of rheumatic autoimmune diseases (34), vs. 56% co-occurrence with hEDS/HSD in this study. The more severe disease (more comorbidities) was observed regardless of whether the patient was diagnosed with hEDS&Fibro or HSD&Fibro; however, most patients were diagnosed with HSD (76.6%) vs. hEDS (23.4%) so HSD&Fibro was more common in our study.

Around 13% of patients only had fibromyalgia, but we did not assess whether they were older women post-menopause that may not have met the diagnostic criteria for localized or historical HSD because of increased joint stiffness with age. Additionally, patients may experience hypermobility in other locations than those assessed by the Beighton criteria and so not meet the 2017 hypermobile hEDS/HSD diagnostic criteria. If this were the case, the co-occurrence of fibromyalgia with hypermobile syndromes may be as high as 70%. It is also possible that fibromyalgia may occur as a primary diagnosis in patients in this study that were not diagnosed with hEDS/HSD. A number of other studies have reported co-occurrence of fibromyalgia with hEDS/HSD but did not study a large number of patients or a large number of symptoms/comorbidities (16–18, 35, 36).

Around 90–95% of fibromyalgia and hEDS/HSD patients in this study were White females, like other studies (1, 2). Because most patients were women, we analyzed the data removing men, but found that there were, in general, no important differences in the results. There were too few men in the cohort to compare sex differences, but this is an area that will likely yield mechanistic and therapeutic insight and should be examined in the future.

Another important finding of the study is that around 30–35% of patients with fibromyalgia ± hEDS/HSD reported PTSD and/or a history of abuse (verbal, physical or sexual), compared to only 10–15% of hEDS/HSD patients. We did not examine the age of abuse in this study, but numerous studies have shown that physical and mental trauma in childhood (i.e., childhood adverse experiences/ACEs) significantly increases the risk of developing mental health issues like anxiety and depression and chronic diseases like autoimmune disease later in life (37–40). Early life stressors alter the hypothalamic-pituitary axis and neurobiology creating central and peripheral nerve hypersensitivity to painful and other stimuli leading to central sensitization- an important factor contributing to pain in fibromyalgia and hEDS/HSD patients (2, 39, 41).

Persistent chronic pain conditions like fibromyalgia, myalgic encephalomyelitis (ME)/chronic fatigue syndrome (CFS), IBS and TMJ are defined as functional somatic syndromes where symptoms cannot be explained by physical damage or disease and a psychological explanation is proposed (42, 43). Our study found that the majority of hEDS/HSD and fibromyalgia patients self-reported subluxations, dislocations (hEDS) and other joint issues like TMJ. Clinical medicine does not currently have good imaging methods or tests to detect extracellular matrix damage and scar formation (i.e., fibrosis, adhesions) unless they occur within an organ. From a physiological and immunological perspective, subluxations caused by hypermobility in hEDS/HSD patients may lead to tissue/matrix damage by releasing proteins and other mediators like extracellular vesicles that activate resident mast cells and macrophages and resident tissue cells like fibroblasts that are present in the joint matrix (15–17, 40). TMJ is often considered to be due to stress from clenching/grinding teeth, but in patients with hEDS/HSD it often results from joint instability, subluxation and/or dislocation- a potential physical cause of tissue damage. More recently, the central role of the immune system, and especially mast cells, in tri-communication with the immune, nervous and hormone/stress systems has led researchers to discover changes in inflammation and inflammatory biomarkers in these conditions (e.g., IBS, migraine) that directly influence pain and nerve function [reviewed in Meade and Garvey (40)], providing a physical rather than purely psychological explanation for symptoms (40, 44). Mast cells directly interact with the nervous system where they can promote pain via release of substance P and other mediators like pain-enhancing cytokines such as tumor necrosis factor (TNF) and IL-1β (24, 40, 44–47). More research is needed to characterize the immune response in patients with fibromyalgia and hEDS/HSD, but in this study the majority of fibromyalgia (and hEDS/HSD) patients reported subluxations, dislocations and other joint instabilities that could be a form of constant physical damage providing both a physical cause for symptoms and a potential source of mast cell activation. Therapeutic approaches that stabilize joints and reduce mast cell activation (e.g., reduce food allergies and intolerances and joint instability) and other inflammation (44, 47) in conjunction with education and cognitive behavioral therapy (48) may benefit fibromyalgia as well as hEDS/HSD patients.

Although there was extensive symptom/comorbidity overlap between patient groups, we found that all 40 self-reported issues that we examined occurred significantly less often in hEDS/HSD patients that did not also have fibromyalgia than in patients with fibromyalgia. Additionally, we found 5 symptoms/comorbidities that were significantly and uniquely worse in patients with hEDS/HSD plus Fibro including subluxations, joint issues like sprains, the need to stop sports due to injuries, poor wound healing, and migraine (Figure 1). In patients with hEDS&Fibro that list also included dislocations. In contrast, the top issues in patients with fibromyalgia regardless of the presence of hEDS/HSD were joint pain, hand pain when writing or typing, brain fog, joint pain keeping from daily activities, allergy/atopy, and headache (Figure 1). This dichotomy of symptoms represents “classic” hEDS/HSD vs. fibromyalgia symptoms and suggests that what we have considered as the “classic” hEDS/HSD patient also has fibromyalgia, which has not been previously recognized. Because 76.6% of the patients diagnosed in our EDS Clinic in this study had HSD, HSD&Fibro represents the majority of patients with the more severe disease phenotype based on many symptoms/comorbidities.

A key finding in this study is that migraine is a primary neurologic self-reported issue in hEDS/HSD plus Fibro patients vs. headaches in fibromyalgia patients. However, with self-reported data we do not know whether the “headache” reported by patients might actually be migraine. Hypermobility is known to be an important contributor to the development of new daily headache (30, 49). Our data suggest that the presence of both fibromyalgia and hEDS/HSD is needed to significantly promote migraine, but it is also true that a diagnosis of migraine may promote fibromyalgia which is supported by the observation that many patients present with migraine/chronic migraine before obtaining a diagnosis of fibromyalgia. If hEDS/HSD contributes to the development of fibromyalgia, this could be another explanation for patients presenting with migraine before being diagnosed with fibromyalgia. Future research is needed to confirm these findings.

There are several limitations to the study. The study site is a tertiary care center and the findings from this cohort of patients may not represent other sites or other regions of the US or world. A strength of the study is that hEDS/HSD was diagnosed using the latest 2017 criteria by a physician. Fibromyalgia was diagnosed using the 2016 revised fibromyalgia diagnostic criteria using a questionnaire that was then confirmed by a physician in-person. However, symptoms and comorbidities were self-reported and were not validated by another method like examining ICD codes or physician diagnoses. This is critical because many issues need clarification like patient reported abnormal MRIs could be incidental or pathologic and self-reported headache could be migraine. Future studies should examine key findings from this study using confirmatory methods and to determine true incident rates of comorbidities. This study did not examine key features of fibromyalgia including fatigue or muscle issues but focused on joint issues and other comorbidities. An additional strength of the study is a large study population with the presence of an internal control group that underwent the same diagnosis process but was not diagnosed with hEDS/HSD or fibromyalgia. Importantly, the control group is not “healthy” but has many symptoms and comorbidities, but these were significantly different than the hEDS/HSD and fibromyalgia patients indicating that they were appropriate controls.

## Data availability statement

The datasets presented in this article are not readily available because of patient privacy. Requests to access the datasets should be directed to DK, knight.dacre@mayo.edu.

## Ethics statement

The retrospective studies involving human participants were reviewed and approved by the Mayo Clinic Institutional Review Board (IRB# 19-011260). Written informed consent for participation was not required for this study in accordance with the national legislation and the institutional requirements.

## Author contributions

DF, KB, and DK agree to be accountable to all aspects of the work ensuring that questions related to accuracy and integrity of any part of the work are properly investigated and resolved, and contributed to the conceptualization and design of the work and wrote the manuscript. DK diagnosed hEDS/HSD and fibromyalgia patients. DF, KB, AD, AK, and AJ built and/or managed the electronic database of patient data. DF, KB, AD, PM, ZP, DH, and DK analyzed the patient data. All authors interpreted the data, edited and revised the manuscript for important content, and approved the final version of the manuscript.
