# Supplementary material for: High overlap in patients diagnosed with hypermobile Ehlers-Danlos syndrome or hypermobile spectrum disorders with fibromyalgia and 40 self-reported symptoms and comorbidities
Source: Front Med (Lausanne). 2023 Apr 25;10:1096180. doi: 10.3389/fmed.2023.1096180 (PMC10166812; doi:10.3389/fmed.2023.1096180)
Supplement: Supplementary file 1 [file Table_1.DOCX]

Supplementary Material

# Supplementary Data

Supplemental Table 1. Comparison of female and male patients diagnosed with Fibromyalgia (Fibro) only (*n* = 98) or hEDS/HSD only (*n* =167) or hEDS/HSD and Fibro (*n* = 414) vs. controls with no Fibro or hEDS/HSD (n =54) to corresponding controls

| Condition | Control^a^ (no Fibro, hEDS/ HSD)  (*n* = 54) | Control (Fibro only)  (*n* = 98) | hEDS/HSD only  (*n* = 167) | hEDS/ HSD/ Fibro  (*n* = 414) | Total  (*n*=733) | P value^b^ |
| --- | --- | --- | --- | --- | --- | --- |
| *Allergy/Atopy* |  |  |  |  |  | 0.002 |
| Missing | 7 | 10 | 15 | 42 | 74 |  |
| Yes | 31 (66.0%) | 68 (77.3%) | 106 (69.7%) | 303 (81.5%) | 508 (77.1%) |  |
| No | 15 (31.9%) | 14 (15.9%) | 41 (27.0%) | 48 (12.9%) | 118 (17.9%) |  |
| Unknown | 1 (2.1%) | 6 (6.8%) | 5 (3.3%) | 21 (5.6%) | 33 (5.0%) |  |
| *Rhinitis/hay fever* |  |  |  |  |  | <0.001 |
| Yes | 19 (35.2%) | 52 (53.1%) | 52 (31.1%) | 186 (44.9%) | 309 (42.2%) |  |
| No | 35 (64.8%) | 46 (46.9%) | 115 (68.9%) | 228 (55.1%) | 424 (57.8%) |  |
| *Previous hypermobile assessment* |  |  |  |  |  | <0.001 |
| Yes | 16 (29.6%) | 40 (40.8%) | 71 (42.5%) | 255 (61.6%) | 382 (52.1%) |  |
| No | 33 (61.1%) | 48 (49.0%) | 89 (53.3%) | 144 (34.8%) | 314 (42.8%) |  |
| Unknown | 5 (9.3%) | 10 (10.2%) | 7 (4.2%) | 15 (3.6%) | 37 (5.0%) |  |
| *Stop sports due to injury?* |  |  |  |  |  | <0.001 |
| Missing | 12 | 31 | 33 | 75 | 151 |  |
| Yes | 22 (52.4%) | 26 (38.8%) | 76 (56.7%) | 251 (74.0%) | 375 (64.4%) |  |
| No | 20 (47.6%) | 41 (61.2%) | 58 (43.3%) | 88 (26.0%) | 207 (35.6%) |  |
| *Worse after stopping?* |  |  |  |  |  | <0.001 |
| Missing | 12 | 31 | 33 | 75 | 151 |  |
| Yes | 14 (33.3%) | 37 (55.2%) | 56 (41.8%) | 219 (64.6%) | 326 (56.0%) |  |
| No | 23 (54.8%) | 30 (44.8%) | 66 (49.3%) | 110 (32.4%) | 229 (39.3%) |  |
| Still involved | 5 (11.9%) | 0 (0.0%) | 12 (9.0%) | 10 (2.9%) | 27 (4.6%) |  |
| *Previously diagnosed with hypermobile syndrome* | 4 (7.4%) | 16 (16.3%) | 17 (10.2%) | 58 (14.0%) | 95 (13.0%) | 0.28 |
| *Previously diagnosed with HSD* | 3 (5.6%) | 5 (5.1%) | 7 (4.2%) | 24 (5.8%) | 39 (5.3%) | 0.89 |
| *Previously diagnosed with hEDS* | 4 (7.4%) | 14 (14.3%) | 29 (17.4%) | 151 (36.5%) | 198 (27.0%) | <0.001 |
| *Previously diagnosed with general EDS* | 1 (1.9%) | 9 (9.2%) | 5 (3.0%) | 21 (5.1%) | 36 (4.9%) | 0.14 |
| *Previously diagnosed with other types of EDS* | 0 (0.0%) | 5 (5.1%) | 3 (1.8%) | 12 (2.9%) | 20 (2.7%) | 0.305 |
| *No previous diagnosis* | 4 (7.4%) | 4 (4.1%) | 13 (7.8%) | 32 (7.7%) | 53 (7.2%) | 0.643 |
| *Unknown* | 2 (3.7%) | 2 (2.0%) | 8 (4.8%) | 21 (5.1%) | 33 (4.5%) | 0.657 |
| *Family member with similar diagnosis* |  |  |  |  |  | <0.001 |
| Yes | 18 (33.3%) | 43 (43.9%) | 94 (56.3%) | 276 (66.7%) | 431 (58.8%) |  |
| No | 32 (59.3%) | 37 (37.8%) | 57 (34.1%) | 96 (23.2%) | 222 (30.3%) |  |
| Unknown | 4 (7.4%) | 18 (18.4%) | 16 (9.6%) | 42 (10.1%) | 80 (10.9%) |  |
| *Clumsy* |  |  |  |  |  | <0.001 |
| Yes | 24 (44.4%) | 75 (76.5%) | 100 (59.9%) | 357 (86.2%) | 556 (75.9%) |  |
| No | 27 (50.0%) | 22 (22.4%) | 65 (38.9%) | 48 (11.6%) | 162 (22.1%) |  |
| Unknown | 3 (5.6%) | 1 (1.0%) | 2 (1.2%) | 9 (2.2%) | 15 (2.0%) |  |
| *Joint pain* | 37 (68.5%) | 95 (96.9%) | 139 (83.2%) | 403 (97.3%) | 674 (92.0%) | < 0.001 |
| *Subluxations* | 24 (44.4%) | 63 (64.3%) | 110 (65.9%) | 336 (81.2%) | 533 (72.7%) | <0.001 |
| *Dislocations* | 13 (24.1%) | 25 (25.5%) | 42 (25.1%) | 153 (37.0%) | 233 (31.8%) | 0.012 |
| *Sprains* | 24 (44.4%) | 59 (60.2%) | 97 (58.1%) | 325 (78.5%) | 505 (68.9%) | <0.001 |
| *None* | 11 (20.4%) | 1 (1.0%) | 7 (4.2%) | 5 (1.2%) | 24 (3.3%) | <0.001 |
| *Joint pain keeping from daily activities* |  |  |  |  |  | <0.001 |
| Missing | 11 | 1 | 7 | 5 | 24 |  |
| Yes | 26 (60.5%) | 86 (88.7%) | 94 (58.8%) | 364 (89.0%) | 570 (80.4%) |  |
| No | 17 (39.5%) | 11 (11.3%) | 66 (41.2%) | 45 (11.0%) | 139 (19.6%) |  |
| *Hand pain after writing/ typing* |  |  |  |  |  | <0.001 |
| Missing | 11 | 1 | 7 | 5 | 24 |  |
| Yes | 31 (72.1%) | 87 (89.7%) | 114 (71.2%) | 374 (91.4%) | 606 (85.5%) |  |
| No | 12 (27.9%) | 10 (10.3%) | 46 (28.8%) | 35 (8.6%) | 103 (14.5%) |  |
| *Jaw clicking, TMJ* |  |  |  |  |  | <0.001 |
| Yes | 35 (64.8%) | 74 (75.5%) | 103 (61.7%) | 363 (87.7%) | 575 (78.4%) |  |
| No | 18 (33.3%) | 17 (17.3%) | 55 (32.9%) | 41 (9.9%) | 131 (17.9%) |  |
| Unknown | 1 (1.9%) | 7 (7.1%) | 9 (5.4%) | 10 (2.4%) | 27 (3.7%) |  |
| *Broken bones* |  |  |  |  |  | 0.045 |
| Yes | 31 (57.4%) | 61 (62.2%) | 83 (49.7%) | 255 (61.6%) | 430 (58.7%) |  |
| No | 22 (40.7%) | 37 (37.8%) | 79 (47.3%) | 143 (34.5%) | 281 (38.3%) |  |
| Unknown | 1 (1.9%) | 0 (0.0%) | 5 (3.0%) | 16 (3.9%) | 22 (3.0%) |  |
| *History of significant surgeries on joints, bones or ligaments* |  |  |  |  |  | 0.636 |
| Yes | 17 (31.5%) | 36 (36.7%) | 52 (31.1%) | 160 (38.6%) | 265 (36.2%) |  |
| No | 36 (66.7%) | 60 (61.2%) | 112 (67.1%) | 243 (58.7%) | 451 (61.5%) |  |
| Unknown | 1 (1.9%) | 2 (2.0%) | 3 (1.8%) | 11 (2.7%) | 17 (2.3%) |  |
| *Joint pain worse during menses?* |  |  |  |  |  | <0.001 |
| Missing | 12 | 9 | 21 | 27 | 69 |  |
| Same | 7 (16.7%) | 11 (12.4%) | 36 (24.7%) | 60 (15.5%) | 114 (17.2%) |  |
| Worse | 8 (19.0%) | 50 (56.2%) | 46 (31.5%) | 212 (54.8%) | 316 (47.6%) |  |
| Unknown | 27 (64.3%) | 28 (31.5%) | 64 (43.8%) | 115 (29.7%) | 234 (35.2%) |  |
| *Headache* | 19 (35.2%) | 74 (75.5%) | 101 (60.5%) | 335 (80.9%) | 529 (72.2%) | < 0.001 |
| *Daily persistent headache* | 4 (7.4%) | 32 (32.7%) | 27 (16.2%) | 158 (38.2%) | 221 (30.2%) | < 0.001 |
| *Cluster headache* | 5 (9.3%) | 10 (10.2%) | 6 (3.6%) | 71 (17.1%) | 92 (12.6%) | < 0.001 |
| *Migraine* | 20 (37.0%) | 42 (42.9%) | 71 (42.5%) | 264 (63.8%) | 397 (54.2%) | < 0.001 |
| *Chronic migraine* | 10 (18.5%) | 29 (29.6%) | 23 (13.8%) | 141 (34.1%) | 203 (27.7%) | < 0.001 |
| *Chiari malformation* | 1 (1.9%) | 8 (8.2%) | 0 (0.0%) | 27 (6.5%) | 36 (4.9%) | < 0.001 |
| *Intracranial hypertension* | 1 (1.9%) | 5 (5.1%) | 2 (1.2%) | 28 (6.8%) | 36 (4.9%) | 0.018 |
| *CSF leak* | 1 (1.9%) | 3 (3.1%) | 1 (0.6%) | 18 (4.3%) | 23 (3.1%) | 0.087 |
| *Current or past abnormal brain MRI* | 1 (1.9%) | 17 (17.3%) | 8 (4.8%) | 59 (14.3%) | 85 (11.6%) | < 0.001 |
| *Autonomic dysfunction* | 6 (11.1%) | 39 (39.8%) | 18 (10.8%) | 157 (37.9%) | 220 (30.0%) | < 0.001 |
| *Neuropathy* | 7 (13.0%) | 44 (44.9%) | 28 (16.8%) | 134 (32.4%) | 213 (29.1%) | < 0.001 |
| *Vertigo* | 11 (20.4%) | 38 (38.8%) | 31 (18.6%) | 189 (45.7%) | 269 (36.7%) | < 0.001 |
| *Brain Fog* | 22 (40.7%) | 87 (88.8%) | 82 (49.1%) | 369 (89.1%) | 560 (76.4%) | < 0.001 |
| *Tinnitus* | 12 (22.2%) | 57 (58.2%) | 49 (29.3%) | 227 (54.8%) | 345 (47.1%) | < 0.001 |
| *Autism/ ASD* | 1 (1.9%) | 5 (5.1%) | 4 (2.4%) | 24 (5.8%) | 34 (4.6%) | 0.273 |
| *Bruise Easily* |  |  |  |  |  | < 0.001 |
| Yes | 28 (51.9%) | 80 (81.6%) | 122 (73.1%) | 353 (85.3%) | 583 (79.5%) |  |
| No | 26 (48.1%) | 18 (18.4%) | 45 (26.9%) | 61 (14.7%) | 150 (20.5%) |  |
| *Poor wound healing* |  |  |  |  |  | < 0.001 |
| Yes | 19 (35.2%) | 54 (55.1%) | 58 (34.7%) | 288 (69.6%) | 419 (57.2%) |  |
| No | 35 (64.8%) | 44 (44.9%) | 109 (65.3%) | 126 (30.4%) | 314 (42.8%) |  |
| *Easy scarring* |  |  |  |  |  | < 0.001 |
| Yes | 21 (38.9%) | 67 (68.4%) | 92 (55.1%) | 321 (77.5%) | 501 (68.3%) |  |
| No | 33 (61.1%) | 31 (31.6%) | 75 (44.9%) | 93 (22.5%) | 232 (31.7%) |  |
| *IBS* | 10 (18.5%) | 46 (46.9%) | 44 (26.3%) | 172 (41.5%) | 272 (37.1%) | < 0.001 |
| *Crohn’s disease* | 2 (3.7%) | 1 (1.0%) | 0 (0.0%) | 11 (2.7%) | 14 (1.9%) | 0.070 |
| *Ulcerative colitis* | 3 (5.6%) | 5 (5.1%) | 0 (0.0%) | 8 (1.9%) | 16 (2.2%) | 0.006 |
| *Constipation* | 17 (31.5%) | 63 (64.3%) | 72 (43.1%) | 264 (63.8%) | 416 (56.8%) | < 0.001 |
| *Diarrhea* | 12 (22.2%) | 56 (57.1%) | 56 (33.5%) | 258 (62.3%) | 382 (52.1%) | < 0.001 |
| *Nausea* | 16 (29.6%) | 69 (70.4%) | 51 (30.5%) | 317 (76.6%) | 453 (61.8%) | < 0.001 |
| *Vomiting* | 9 (16.7%) | 31 (31.6%) | 17 (10.2%) | 179 (43.2%) | 236 (32.2%) | < 0.001 |
| *Heartburn* | 8 (14.8%) | 39 (39.8%) | 28 (16.8%) | 169 (40.8%) | 244 (33.3%) | < 0.001 |
| *Anxiety* | 28 (51.9%) | 65 (66.3%) | 82 (49.1%) | 311 (75.1%) | 486 (66.3%) | < 0.001 |
| *Depression* | 16 (29.6%) | 58 (59.2%) | 61 (36.5%) | 249 (60.1%) | 384 (52.4%) | < 0.001 |
| *PTSD* | 3 (5.6%) | 31 (31.6%) | 18 (10.8%) | 136 (32.9%) | 188 (25.6%) | < 0.001 |
| *Have you been abused?* |  |  |  |  |  | < 0.001 |
| Yes | 10 (18.5%) | 34 (34.7%) | 28 (16.8%) | 148 (35.7%) | 220 (30.0%) |  |
| No | 40 (74.1%) | 54 (55.1%) | 122 (73.1%) | 199 (48.1%) | 415 (56.6%) |  |
| Unknown | 1 (1.9%) | 1 (1.0%) | 3 (1.8%) | 10 (2.4%) | 15 (2.0%) |  |
| Choose not to disclose | 3 (5.6%) | 9 (9.2%) | 14 (8.4%) | 57 (13.8%) | 83 (11.3%) |  |
| *Verbal/ emotional abuse* | 9 (16.7%) | 28 (28.6%) | 20 (12.0%) | 123 (29.7%) | 180 (24.6%) | < 0.001 |
| *Physical abuse* | 7 (13.0%) | 18 (18.4%) | 10 (6.0%) | 80 (19.3%) | 115 (15.7%) | < 0.001 |
| *Sexual abuse* | 3 (5.6%) | 22 (22.4%) | 13 (7.8%) | 93 (22.5%) | 131 (17.9%) | < 0.001 |
| *Unknown/ chose not to disclose type of abuse* | 0 (0.0%) | 4 (4.1%) | 3 (1.8%) | 4 (1.0%) | 11 (1.5%) | 0.129 |

***^a^*** Abbreviations: ASD, autism spectrum disorder; CSF, cerebral spinal fluid; Fibro, fibromyalgia; hEDS, hypermobile Ehlers-Danlos syndrome; HSD, hypermobility spectrum disorder; IBS, irritable bowel syndrome; MRI, magnetic resonance imaging; PTSD, post-traumatic stress disorder; TMJ, temporomandibular joint dysfunction. ***^b^*** P values result from Fisher's test for categorical data and Kruskal-Wallis rank sum test for numeric data.

Supplementary Table 2. Comparison of symptoms/comorbidities found in female patients diagnosed with fibromyalgia, hypermobility (hEDS or HSD) or hEDS/HSD and fibromyalgia compared to controls (*n* = 664)

| Symptom/ comorbidity | Control (no Fibro, hEDS/ HSD) (*n* = 42) | Fibro only  (*n* = 89) | hEDS/HSD only  (*n* = 146) | hEDS/ HSD & Fibro  (*n* = 387) | *P* value |
| --- | --- | --- | --- | --- | --- |
| Past hypermobile assessment? | 13 (31.0%) | 37 (41.6%) | 65 (44.5%) | 237 (61.2%)*** | < 0.001 |
| Previously diagnosed with hEDS | 4 (9.5%) | 12 (13.5%) | 28 (19.2%) | 141 (36.4%)*** | < 0.001 |
| Previously diagnosed with HSD | 2 (4.8%) | 4 (4.5%) | 6 (4.1%) | 21 (5.4%) | 0.98 |
| Previously diagnosed with general EDS? | 1 (2.4%) | 9 (10.1%) | 3 (2.1%) | 21 (5.4%) | 0.06 |
| Previously diagnosed with other type of EDS? | 0 (0.0%) | 5 (5.6%) | 3 (2.1%) | 11 (2.8%) | 0.32 |
| No previous EDS diagnosis | 3 (7.1%) | 3 (3.4%) | 11 (7.5%) | 28 (7.2%) | 0.58 |
| Family member with similar diagnosis | 16 (38.1%) | 39 (43.8%) | 83 (56.8%)* | 259 (66.9%)*** | < 0.001 |
| Clumsy | 23 (54.8%) | 69 (77.5%)* | 93 (63.7%) | 334 (86.3%)*** | < 0.001 |
| Joint pain | 30 (71.4%) | 86 (96.6%)*** | 124 (84.9%) | 377 (97.4%)*** | < 0.001 |
| Joint pain worse during menses? | 8 (19.0%) | 50 (56.2%) | 46 (31.5%) | 212 (54.8%) | < 0.001 |
| Do you have hand pain after writing or typing? | 27 (77.1%) | 80 (90.9%) | 99 (70.7%) | 348 (90.9%)* | < 0.001 |
| Jaw click/ TMJ | 30 (71.4%) | 70 (78.7%) | 94 (64.4%) | 341 (88.1%)** | < 0.001 |
| Joint issues? e.g., Sprains | 20 (47.6%) | 56 (62.9%) | 88 (60.3%) | 306 (79.1%)*** | < 0.001 |
| Subluxation | 21 (50.0%) | 56 (62.9%) | 97 (66.4%) | 315 (81.4%)*** | < 0.001 |
| Dislocations | 9 (21.4%) | 23 (25.8%) | 34 (23.3%) | 138 (35.7%) | 0.01 |
| No joint issues | 7 (16.7%) | 1 (1.1%)** | 6 (4.1%)* | 4 (1.0%)*** | < 0.001 |
| Were you involved in sports that required flexibility (e.g., gymnastics, dance) | 24 (57.1%) | 45 (50.6%) | 90 (61.6%) | 246 (63.6%) | 0.30 |
| Stop sports due to injury? | 16 (50.0%) | 24 (38.7%) | 67 (58.3%) | 231 (73.3%)** | < 0.001 |
| Worse after stopped sports? | 11 (34.4%) | 33 (53.2%)** | 52 (45.2%) | 206 (65.4%)*** | < 0.001 |
| Does joint pain keep you from daily activities? | 19 (54.3%) | 81 (92.0%)*** | 85 (60.7%) | 341 (89.0%)*** | < 0.001 |
| Scoliosis | 14 (33.3%) | 36 (40.4%) | 39 (26.7%) | 145 (37.5%) | 0.02 |
| Have you ever broken a bone? | 22 (52.4%) | 55 (61.8%) | 72 (49.3%) | 239 (61.8%) | 0.05 |
| History of significant surgeries on joints, bones or ligaments? | 12 (28.6%) | 32 (36.0%) | 46 (31.5%) | 149 (38.5%) | 0.62 |
| Easily bruised | 26 (61.9%) | 76 (85.4%)** | 115 (78.8%)* | 334 (86.3%)*** | 0.002 |
| History of easy scarring | 20 (47.6%) | 62 (69.7%)* | 83 (56.8%) | 302 (78.0%)*** | < 0.001 |
| Poor wound healing | 18 (42.9%) | 49 (55.1%) | 51 (34.9%) | 267 (69.0%)*** | < 0.001 |
| Allergy/ Atopy | 29 (80.6%) | 61 (77.2%) | 98 (72.1%) | 285 (81.9%) | 0.045 |
| Hayfever | 17 (40.5%) | 47 (52.8%) | 50 (34.2%) | 177 (45.7%) | 0.03 |
| Headache | 18 (42.9%) | 67 (75.3%)*** | 90 (61.6%)* | 314 (81.1%)*** | < 0.001 |
| Daily persistent headache | 4 (9.5%) | 30 (33.7%)** | 24 (16.4%) | 147 (38.0%)*** | < 0.001 |
| Cluster headache | 5 (11.9%) | 9 (10.1%) | 5 (3.4%)* | 64 (16.5%) | < 0.001 |
| Migraine | 19 (45.2%) | 41 (46.1%) | 64 (43.8%) | 249 (64.3%)* | < 0.001 |
| Chronic migraine | 9 (21.4%) | 28 (31.5%) | 22 (15.1%) | 135 (34.9%) | < 0.001 |
| Chiari malformation | 1 (2.4%) | 8 (9.0%) | 0 (0.0%) | 24 (6.2%) | 0.001 |
| Intracranial hypertension | 1 (2.4%) | 5 (5.6%) | 2 (1.4%) | 26 (6.7%) | 0.06 |
| CSF leak | 1 (2.4%) | 3 (3.4%) | 1 (0.7%) | 16 (4.1%) | 0.19 |
| Current or past abnormal brain MRI? | 1 (2.4%) | 17 (19.1%)* | 8 (5.5%) | 57 (14.7%)* | 0.001 |
| Autonomic dysfunction | 5 (11.9%) | 35 (39.3%)** | 17 (11.6%) | 147 (38.0%)*** | < 0.001 |
| Neuropathy | 5 (11.9%) | 42 (47.2%)*** | 28 (19.2%) | 127 (32.8%)** | < 0.001 |
| Vertigo | 9 (21.4%) | 36 (40.4%)* | 30 (20.5%) | 177 (45.7%)** | < 0.001 |
| Brain fog | 21 (50.0%) | 78 (87.6%)*** | 74 (50.7%) | 344 (88.9%)*** | < 0.001 |
| Tinnitus | 10 (23.8%) | 50 (56.2%)*** | 44 (30.1%) | 214 (55.3%)*** | < 0.001 |
| Nausea | 16 (38.1%) | 62 (69.7%)** | 47 (32.2%) | 295 (76.2%)*** | < 0.001 |
| Heartburn | 7 (16.7%) | 36 (40.4%)** | 26 (17.8%) | 162 (41.9%)** | < 0.001 |
| Vomiting | 9 (21.4%) | 29 (32.6%) | 16 (11.0%) | 168 (43.4%)** | < 0.001 |
| Diarrhea | 10 (23.8%) | 51 (57.3%)*** | 49 (33.6%) | 239 (61.8%)*** | < 0.001 |
| Constipation | 17 (40.5%) | 60 (67.4%)** | 68 (46.6%) | 252 (65.1%)** | < 0.001 |
| IBS | 9 (21.4%) | 45 (50.6%)** | 42 (28.8%) | 159 (41.1%)* | < 0.001 |
| Crohn’s disease | 1 (2.4%) | 1 (1.1%) | 0 (0.0%) | 9 (2.3%) | 0.19 |
| Ulcerative colitis | 2 (4.8%) | 5 (5.6%) | 0 (0.0%)* | 7 (1.8%) | 0.01 |
| Autism/ ASD | 1 (2.4%) | 4 (4.5%) | 2 (1.4%) | 17 (4.4%) | 0.36 |
| Anxiety | 25 (59.5%) | 62 (69.7%) | 73 (50.0%) | 290 (74.9%)* | < 0.001 |
| Depression | 14 (33.3%) | 54 (60.7%)** | 52 (35.6%) | 230 (59.4%)** | < 0.001 |
| PTSD | 3 (7.1%) | 30 (33.7%)*** | 17 (11.6%) | 127 (32.8%)*** | < 0.001 |
| Have you ever been abused? | 10 (23.8%) | 33 (37.1%) | 25 (17.1%) | 140 (36.2%) | < 0.001 |
| Emotional abuse | 9 (21.4%) | 27 (30.3%) | 17 (11.6%) | 116 (30.0%) | < 0.001 |
| Physical abuse | 7 (16.7%) | 17 (19.1%) | 8 (5.5%) | 76 (19.6%) | < 0.001 |
| Sexual abuse | 3 (7.1%) | 21 (23.6%)* | 12 (8.2%) | 91 (23.5%)* | < 0.001 |
| Unknown/ chose not to disclose abuse | 0 (0.0%) | 4 (4.5%) | 3 (2.1%) | 3 (0.8%) | 0.06 |

***^a^*** Abbreviations: CSF, cerebral spinal fluid; Fibro, fibromyalgia; hEDS, hypermobile Ehlers-Danlos syndrome; HSD, hypermobility spectrum disorder; IBS, irritable bowel syndrome; MRI, magnetic resonance imaging; TMJ; temporomandibular joint dysfunction. ***^b^*** P values result from Fisher's test for categorical data and Kruskal-Wallis rank sum test for numeric data. Post-hoc analysis compares Fibro, hEDS/HSD or hEDS/HSD&Fibro to control patients that were not diagnosed with hypermobility or fibromyalgia: *, *p* < 0.05; **, *p* < 0.01; ***, *p* < 0.001.

Supplementary Table 3. Summary of conditions self-reported in female and male patients diagnosed with fibromyalgia (*n* = 98)

| Conditions*^a^* | Fibromyalgia |
| --- | --- |
| Joint pain | 96.9% |
| Hand pain with writing/ typing | 89.7% |
| Brain fog | 88.8% |
| Joint pain keeps from daily activities | 88.7% |
| Allergy/atopy | 77.3% |
| Clumsy | 76.5% |
| Jaw clicks | 75.5% |
| Headache | 75.5% |
| Nausea | 70.4% |
| History of easy scarring | 68.4% |
| Anxiety | 66.3% |
| Constipation | 64.3% |
| Subluxations | 64.3% |
| Joint issues e.g., sprains | 60.2% |
| Depression | 59.2% |
| Tinnitus | 58.2% |
| Diarrhea | 57.1% |
| Joint pain with menses | 56.2% |
| Poor wound healing | 55.1% |
| Worse joint problems after stopping sports | 55.2% |
| Hayfever | 53.1% |
| Neuropathy | 44.9% |
| Family member with similar diagnosis | 43.9% |
| Migraine | 42.9% |
| Scoliosis | 40.8% |
| Heartburn | 39.8% |
| Autonomic dysfunction | 39.8% |
| Stop sports due to injury | 38.8% |
| Vertigo | 38.8% |
| History of abuse | 34.7% |
| Daily persistent headache | 32.7% |
| Vomiting | 31.6% |
| PTSD | 31.6% |
| Chronic migraine (>8 days/ month) | 29.6% |
| Emotional abuse | 28.6% |
| Sexual abuse | 22.4% |
| Physical abuse | 18.4% |

***^a^*** Order of conditions based on highest to lowest % in patients diagnosed with fibromyalgia.

Supplementary Table 4. Summary of conditions self-reported in female and male patients diagnosed with hEDS or HSD (*n* = 167)

| Condition*^a^* | hEDS/HSD |
| --- | --- |
| Joint pain | 83.2% |
| Hand pain with writing/ typing | 71.2% |
| Subluxations | 65.9% |
| Jaw clicks | 61.7% |
| Headache | 60.5% |
| Clumsy | 59.9% |
| Joint pain keeps from daily activities | 58.8% |
| Joint issues e.g., sprains | 58.1% |
| Stop sports due to injury | 56.7% |
| Family member with similar diagnosis | 56.3% |
| History of easy scarring | 55.1% |
| Brain fog | 49.1% |
| Anxiety | 49.1% |
| Constipation | 43.1% |
| Migraine | 42.5% |
| Worse joint problems after stopping sports | 41.8% |
| Depression | 36.5% |
| Poor wound healing | 34.7% |
| Diarrhea | 33.5% |
| Joint pain with menses | 31.5% |
| Hayfever | 31.1% |
| Nausea | 30.5% |
| Tinnitus | 29.3% |
| Scoliosis | 25.7% |
| Vertigo | 18.6% |
| Heartburn | 16.8% |
| History of abuse | 16.8% |
| Neuropathy | 16.8% |
| Daily persistent headache | 16.2% |
| Chronic migraine (>8 days/ month) | 13.8% |
| Emotional abuse | 12.0% |
| Autonomic dysfunction | 10.8% |
| PTSD | 10.8% |
| Vomiting | 10.2% |
| Sexual abuse | 7.8% |
| Physical abuse | 6.0% |

***^a^*** Order of conditions based on highest to lowest % in patients diagnosed with hEDS/HSD.

Supplementary Table 5. Summary of 40 conditions self-reported in female patients diagnosed with hEDS/HSD & fibromyalgia (Fibro) (*n* = 387) vs. fibromyalgia (Fibro) (*n* = 89) or hEDS/HSD (*n* = 146)

| Condition*^a^* | hEDS/HSD &Fibro |  | Fibro | P value, adjusted *P* value | hEDS/HSD | P value, adjusted *P* value |
| --- | --- | --- | --- | --- | --- | --- |
| Joint pain | 97.4% |  | 96.6% | *p*=0.72, *p*>0.99 | 84.9% | *p*<0.001, *p*<0.001 |
| Hand pain with writing/ typing | 90.9% |  | 90.9% | *p*>0.99, *p*>0.99 | 70.7% | *p*<0.001, *p*<0.001 |
| Brain fog | 88.9% |  | 87.6% | *p*=0.71, *p*>0.99 | 50.7% | *p*<0.001, *p*<0.001 |
| Joint pain keeps from daily activities | 89.0% |  | 92.0% | *p*=0.56, *p*>0.99 | 60.7% | *p*<0.001, *p*<0.001 |
| *Jaw clicks/ TMJ | 88.1% |  | 78.7% | *p*=0.046, *p*=0.08 | 64.4% | *p*<0.001, *p*<0.001 |
| *Clumsy | 86.3% |  | 77.5% | *p*=0.04, *p*=0.09 | 63.7% | *p*<0.001, *p*<0.001 |
| Easy bruising | 86.3% |  | 85.4% | *p*=0.87, *p*>0.99 | 78.8% | *p*=0.045, *p*=0.09 |
| Allergy/atopy | 81.9% |  | 77.2% | *p*=0.536, *p*>0.99 | 72.1% | *p*=0.004, *p*=0.008 |
| Subluxations | 81.4% |  | 62.9% | *p*<0.001, *p*<0.001 | 66.4% | *p*<0.001, *p*<0.001 |
| Headache | 81.1% |  | 75.3% | *p*=0.24, *p*=0.48 | 61.6% | *p*<0.001, *p*<0.001 |
| Joint issues e.g., sprains | 79.1% |  | 62.9% | *p*=0.002, *p*=0.004 | 60.3% | *p*<0.001, *p*<0.001 |
| History of easy scarring | 78.0% |  | 69.7% | *p*=0.10, *p*=0.20 | 56.8% | *p*<0.001, *p*<0.001 |
| Nausea | 76.2% |  | 69.7% | *p*=0.22, *p*=0.44 | 32.2% | *p*<0.001, *p*<0.001 |
| Anxiety | 74.9% |  | 69.7% | *p*=0.35, *p*=0.70 | 50.0% | *p*<0.001, *p*<0.001 |
| Stop sports due to injury | 73.3% |  | 38.7% | *p*<0.001, *p*<0.001 | 58.3% | *p*=0.003, *p*=0.006 |
| Poor wound healing | 69.0% |  | 55.1% | *p*=0.02, *p*=0.04 | 34.9% | *p*<0.001, *p*<0.001 |
| *Worse joint problems after stopping sports | 65.4% |  | 53.2% | *p*=0.04, *p*=0.08 | 45.2% | *p*<0.001, *p*<0.001 |
| Constipation | 65.1% |  | 67.4% | *p*=0.71, *p*>0.99 | 46.6% | *p*<0.001, *p*<0.001 |
| Migraine | 64.3% |  | 46.1% | *p*=0.002, *p*=0.003 | 43.8% | *p*<0.001, *p*<0.001 |
| Have you ever broken a bone? | 61.8% |  | 61.8% | *p*=0.15, *p*=0.29 | 49.3% | *p*=0.02, *p*=0.04 |
| Diarrhea | 61.8% |  | 57.3% | *p*=0.47, *p*=0.94 | 33.6% | *p*<0.001, *p*<0.001 |
| Depression | 59.4% |  | 60.7% | *p*=0.91, *p*>0.99 | 35.6% | *p*<0.001, *p*<0.001 |
| Joint pain with menses | 54.8% |  | 56.2% | *p*=0.77, *p*>0.99 | 31.5% | *p*<0.001, *p*<0.001 |
| Tinnitus | 55.3% |  | 56.2% | *p*=0.91, *p*>0.99 | 30.1% | *p*<0.001, *p*<0.001 |
| Vertigo | 45.7% |  | 40.4% | *p*=0.41, *p*=0.82 | 20.5% | *p*<0.001, *p*<0.001 |
| Hayfever | 45.7% |  | 52.8% | *p*=0.24, *p*=0.48 | 34.2% | *p*=0.02, *p*=0.04 |
| Vomiting | 43.4% |  | 32.6% | *p*=0.07, *p*=0.15 | 11.0% | *p*<0.001, *p*<0.001 |
| Heartburn | 41.9% |  | 40.4% | *p*=0.91, *p*>0.99 | 17.8% | *p*<0.001, *p*<0.001 |
| IBS | 41.1% |  | 50.6% | *p*=0.12, *p*=0.25 | 28.8% | *p*=0.009, *p*=0.02 |
| Daily persistent headache | 38.0% |  | 33.7% | *p*=0.47, *p*=0.94 | 16.4% | *p*<0.001, *p*<0.001 |
| Autonomic dysfunction | 38.0% |  | 39.3% | *p*=0.81, p>0.99 | 11.6% | *p*<0.001, *p*<0.001 |
| Scoliosis | 37.5% |  | 40.4% | *p*=0.67, *p*>0.99 | 26.7% | *p*=0.001, *p*=0.002 |
| History of abuse | 36.2% |  | 37.1% | *p*=0.79, *p*>0.99 | 17.1% | *p*<0.001, *p*<0.001 |
| Dislocations | 35.7% |  | 25.8% | *p*=0.08, *p*=0.17 | 23.3% | *p*=0.007, *p*=0.01 |
| Chronic migraine (>8 days/ month) | 34.9% |  | 31.5% | *p*=0.62, *p*>0.99 | 15.1% | *p*<0.001, *p*<0.001 |
| PTSD | 32.8% |  | 33.7% | *p*=0.90, *p*>0.99 | 11.6% | *p*<0.001, *p*<0.001 |
| Neuropathy | 32.8% |  | 47.2% | *p*=0.01, *p*=0.03 | 19.2% | *p*=0.002, *p*=0.004 |
| Emotional abuse | 30.0% |  | 30.3% | *p*>0.99, *p*>0.99 | 11.6% | *p*<0.001, *p*<0.001 |
| Sexual abuse | 23.5% |  | 23.6% | *p*>0.99, *p*>0.99 | 8.2% | *p*<0.001, *p*<0.001 |
| Physical abuse | 19.6% |  | 19.1% | *p*>0.99, *p*>0.99 | 5.5% | *p*<0.001, *p*<0.001 |
| Cluster headache | 16.5% |  | 10.1% | *p*=0.14, *p*=0.29 | 3.4% | *p*<0.001, *p*<0.001 |
| Past or current abnormal MRI | 14.7% |  | 19.1% | *p*=0.33, *p*=0.66 | 5.5% | *p*=0.003, *p*=0.006 |

***^a^*** Order of conditions based on highest to lowest % in patients diagnosed with hEDS/HSD&Fibro. ***^b^*** P values compare Fibro control to hEDS/HSD & Fibro patients. ***^c^*** P values compare hEDS/HSD to hEDS/HSD&Fibro patients. ***^d^*** Conditions primarily associated with hEDS/HSD are marked in *bold*. All other conditions occur more often in patients with fibromyalgia (not bold).
